# Families’ Experiences With Family-Focused Web-Based Interventions for Improving Health: Qualitative Systematic Literature Review

**DOI:** 10.2196/58774

**Published:** 2025-01-30

**Authors:** Diana Zhu, Aimee L Dordevic, Zoe E Davidson, Simone Gibson

**Affiliations:** 1 Department of Nutrition Dietetics and Food Monash University Melbourne Australia; 2 School of Clinical Sciences Monash University Melbourne Australia; 3 Monash Centre for Scholarship in Health Education Monash University Melbourne Australia

**Keywords:** eHealth, family based, qualitative, pediatric health, internet, mobile phone

## Abstract

**Background:**

eHealth interventions can favorably impact health outcomes and encourage health-promoting behaviors in children. More insight is needed from the perspective of children and their families regarding eHealth interventions, including features influencing program effectiveness.

**Objective:**

This review aimed to explore families’ experiences with family-focused web-based interventions for improving health.

**Methods:**

Five databases were searched on October 26, 2022—updated on October 24, 2023—for studies reporting qualitative data on participating children or their caregivers’ experiences with web-based programs. Study identification was performed in duplicate and studies were independently appraised for quality. Thematic synthesis was undertaken on qualitative data extracted from the results section of each included article.

**Results:**

Of 5524 articles identified, 28 articles were included. The studies examined the experiences of school-aged children (aged 5-18 years) and their caregivers (mostly mothers) with 26 web-based interventions that were developed to manage 17 different health conditions or influence health-supporting behaviors. Six themes were identified on families’ experiences: connecting with others, agency of learning, program reputability or credibility, program flexibility, meeting participants’ needs regarding program content or delivery, and impact on lifestyle.

**Conclusions:**

Families positively perceived family-focused web-based interventions, finding value in quality connections and experiencing social support; intervention features aligned with behavioral and self-management principles. Key considerations were highlighted for program developers and health care professionals on ways to adapt eHealth elements to meet families’ health-related needs. Continued research examining families’ experiences with eHealth interventions is needed, including the experiences of families from diverse populations and distinguishing the perspectives of children, their caregivers, and other family members, to inform the expansion of family-focused eHealth interventions in health care systems.

**Trial Registration:**

PROSPERO CRD42022363874; https://tinyurl.com/3xxa8enz

## Introduction

### Pediatric Health

A growing number of children are engaging in health risk behaviors or living with a health condition [1-6]. Children’s management of health conditions greatly influences their physical, emotional, and psychosocial growth; development; and well-being as well as their health into adulthood [1,3,7-9].

The family unit, namely children and their immediate family members (ie, caregivers and siblings), plays an important role in shaping children’s health-related behaviors [6-8,10,11]. Children also assume varied degrees of responsibility for their health with age and into adulthood [3,6]. Long-term multicomponent, multidisciplinary interventions incorporating behavioral change and self-management techniques (eg, disease education, goal setting, and self-monitoring) and involving the family unit are recommended for treating or managing childhood health conditions or encouraging health-promoting behaviors in children [3,12]. Such services are traditionally administered in person; offer limited enrollment with strict eligibility criteria; require extensive time from trained health care professionals (eg, physicians, allied health practitioners, and nurses); and have limited accessibility, particularly in areas of lower socioeconomic advantage or regional or remote areas [3,5,13].

### Pediatric eHealth Interventions

Using digital technologies to adapt conventional services to a web-based setting—offering eHealth interventions—has the potential to overcome the limited accessibility and reach of traditional services for treating childhood health conditions or to influence health-supporting behaviors. Studies have suggested that the use of digital technologies (eg, mobile or video communication platforms or websites) allow the continuation of health care when conventional or specialized care, beyond primary care, is unavailable [5,14].

Existing literature has considered the breadth of eHealth interventions delivered through various eHealth modalities. A 2021 systematic review reported that in the last decade and up until the beginning of the COVID-19 pandemic, there has been a surge in eHealth interventions, largely developed to treat mental illnesses and noncommunicable diseases and delivered through telehealth platforms or mobile phones [15]. Another systematic review in 2021 [4] found that eHealth technologies were mostly used for monitoring, tracking, and reporting purposes. Existing research on eHealth interventions for treating or managing specific health conditions or encouraging health-promoting behaviors in children has focused on evaluating program effectiveness [3,9,12,16-18]. Studies have found that such interventions have improved condition-specific outcomes (eg, disease markers, symptom management, and adherence to disease management plans) and health behavior changes in children [3,12,17-20]. Web-based programs were proposed as a potentially favorable type of eHealth intervention for children and their caregivers [4,21].

With an increased number of families with children living with health conditions needing treatment, urgent action is needed to optimize the development and delivery of eHealth interventions for children and families. Although the evidence base on the effectiveness of such interventions is expanding, there is limited research exploring the experiences of eHealth interventions for children and their families. The needs, values, and perceptions of program end users (ie, children and their caregivers) are essential for program development; participants are key informants of their health, including engagement in health-supporting behaviors or medical treatment [22,23]. Understanding their experiences with eHealth interventions can provide valuable insight on the program’s potential to impact the participating child’s health, including on health outcomes overtime, sustainability of changes, design features to maximize program effectiveness, uptake and engagement, and mechanisms underlying children’s and their families’ health. There is a developing body of literature exploring participants’ lived experiences with family-focused eHealth interventions to prevent or treat health conditions in children.

This qualitative systematic review aims to synthesize the viewpoints of children and their families on their experiences with family-focused web-based programs for improving health. Findings from this review will inform the development of eHealth interventions and enhance our understanding of the ability of eHealth interventions to meet the health-related needs of children and their families.

## Methods

The qualitative systematic literature review was conducted and reported in line with the PRISMA (Preferred Reporting Items for Systematic Reviews and Meta-Analyses) and the Enhancing Transparency in Reporting the Synthesis of Qualitative Research statements [24,25]. The review protocol was prospectively registered via PROSPERO (CRD42022363874). The PRISMA checklist for the review is included in Multimedia Appendix 1.

### Eligibility Criteria

Eligibility criteria were determined using the Population, Intervention, Comparator, Outcome and Study design framework.

#### Population

Studies involving the family unit (at least a child aged ≤18 years and a caregiver) were included in this review. There were no limits on study participants regarding gender, ethnic or medical background, and locality.

#### Intervention

Included study interventions were web-based programs targeted at the family unit. A web-based program was defined as an eHealth intervention where the primary component of the program was completed on the web, including web-based modules and activities. The web-based intervention included the active participation of both the participating child and at least one caregiver (ie, the program included activities for both the participating child and caregiver to complete). The eHealth intervention could have been accessed via multiple technological modalities, such as computers, phones, and tablets. Interventions may have included other adjunct eHealth components (eg, a mobile app for monitoring, tracking, or reporting purposes; email; and phone messaging or calls) or health care services (eg, feedback or support from medical and allied health practitioners). eHealth interventions delivered solely through smartphone apps were not included.

Studies on eHealth interventions where the web-based program was used to support conventional face-to-face health care interventions (including telehealth) or where the web-based component was not the primary part of the intervention were excluded. Studies on web-based interventions used as decision-making, screening, or assessment tools, where the program content was delivered primarily through live sessions (eg, videoconferencing sessions), or on programs targeting the caregiver or child exclusively were also excluded.

#### Comparator

A comparator was not specified for this review.

#### Outcomes

Included studies described the child or caregiver’s perceptions of participating in a web-based program (eg, perceptions of the intervention as a whole or specific intervention features; reflections on occurrences or attitudes before, during, or after the intervention) using qualitative methods, such as interviews, focus groups, and open-ended responses retrieved through surveys.

#### Study Design

All study designs were considered for inclusion if they included a qualitative component exploring participants’ experiences and were published in English. Review articles, doctoral theses, and conference abstracts were excluded.

### Search Strategy

Ovid MEDLINE, Ovid Embase, Cochrane Library, Scopus, and CINAHL were searched for articles that met the eligibility criteria in October 2022 and updated in October 2023. Search results were limited to human studies published in English and within the last decade to capture evidence on the most up-to-date eHealth developments and updated versions of web-based interventions.

The search used both keyword and subheading search terms related to families, web-based programs, experiences or perspectives, and qualitative research methods. The search strategy for each database is included in Multimedia Appendix 2.

To test the validity of the search strategy, 3 key articles that met the inclusion criteria were identified [26-28]. The search strategy was developed with senior researchers with experience in pediatric research or interventions and qualitative research and confirmed with a university librarian. The reference lists of included articles were hand searched for additional relevant articles.

### Study Selection and Data Extraction

Retrieved citations were exported into EndNote 20 software (Clarivate). Duplicates were removed and the remaining results were imported into and managed using Covidence (Veritas Health Innovation). Titles and abstracts were screened independently by at least 2 authors (DZ, SG, and ZED), after which full-text articles were screened independently against the eligibility criteria by 2 authors (DZ, SG, and ZED). Disagreements on the inclusion of articles were resolved by consensus.

Data were extracted using a bespoke data extraction template that was piloted by 2 authors (DZ and SG) before data extraction. Information extracted from articles included publication details (authors and year and country of publication); study aim; participant characteristics (participating children’s age health status and the participating caregivers); study design (qualitative methodology and sample size); intervention characteristics (purpose and key features of the intervention and level of guidance or support provided throughout the intervention); and outcomes (themes and representative quotes relating to participants’ experiences). Data were extracted independently by one author (DZ) and confirmed with a second author (SG); 3 papers were selected randomly where SG independently extracted data to compare and confirm the data extraction process.

### Quality Appraisal

Studies were appraised using the Critical Appraisal Skills Programme qualitative checklist [29]. Included studies were independently assessed by one author (DZ) and confirmed with a second author (SG). Three papers were selected at random and independently appraised by both authors, after which assessment results were discussed, discrepancies were addressed, and the first author appraised the remaining papers.

### Data Synthesis

Thematic synthesis [30] was undertaken on the extracted data about participating children and caregivers’ experiences with the intervention using inductive line-by-line coding of the results and representative quotes extracted from the included studies. Four papers were selected at random and independently and manually coded by 2 authors (DZ and SG). Codes were discussed by both authors (DZ and SG) and refined, whereby the coding process was confirmed. The remaining papers were then coded by DZ, where new codes identified throughout the process were discussed and further verified by SG. One author (DZ) then independently developed descriptive categories from these codes that were discussed and critically reviewed regularly between 2 authors (DZ and SG) until a consensus was reached. These descriptive categories were then synthesized into overarching themes.

Individual and collaborative reflexivity were undertaken during the planning and implementation of the review methodology [31]. Authors were dietitians or nutritionists and researchers, with clinical or research experience in pediatric nutrition, weight management or lifestyle programs, or education. Most authors have been developing their knowledge of or capacity in qualitative methodology, and one author has extensive experience with qualitative research. All authors reflected on and acknowledged their personal and professional experiences by engaging in reflexive writing (eg, researcher notes and journaling) or team discussions throughout the review process. Authors collaborated as a team to resolve disagreements during the screening process and when analyzing and reporting the data. During team discussions, authors also communicated their expertise in the review methodology, assumptions made during decision-making, and expectations of results.

## Results

### Search Results and Study Characteristics

**Figure 1:**
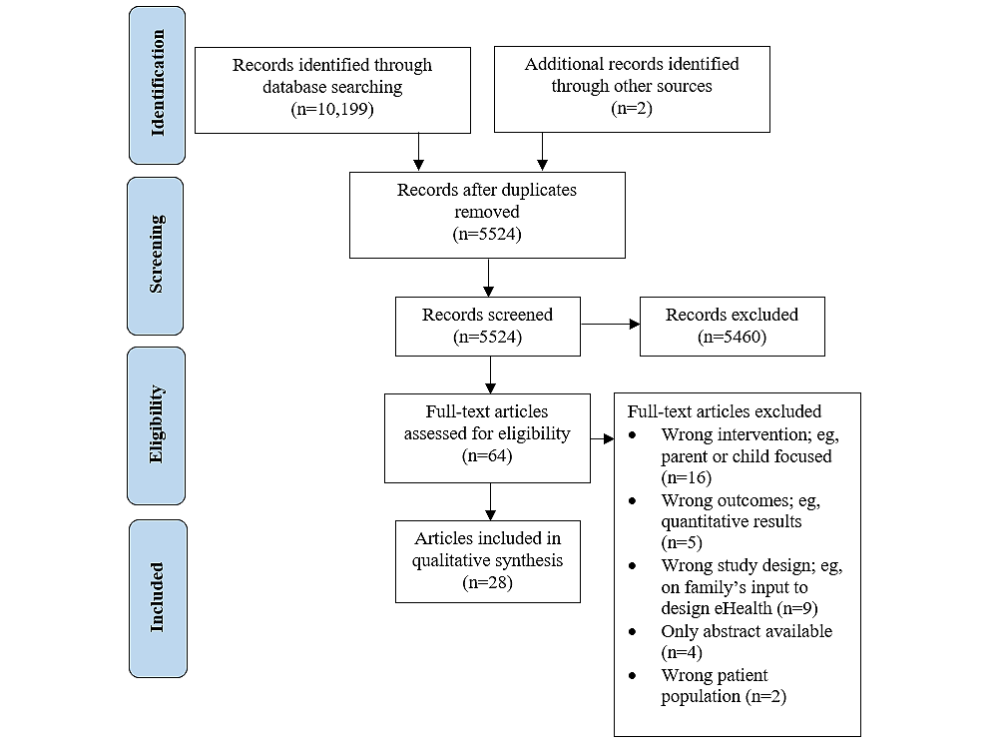
PRISMA (Preferred Reporting Items for Systematic Reviews and Meta-Analyses) flow diagram outlining the study selection process.

The search identified 5524 articles after the removal of duplicates, of which 28 articles were included in the qualitative synthesis (refer to Figure 1 for the PRISMA diagram). Two articles included in this review reported findings from the same study [32,33]. The studies included in this review examined 26 distinct interventions, where 1 study explored 3 versions of an intervention (ie, adapted to 3 cultures) [34], 1 study examined 2 versions of an intervention (ie, partly guided versus entirely self-guided versions) [35], 5 studies evaluated 2 separate interventions (ie, including the conductance of process evaluations on the web-based program) [26,27,36-38], and the remaining studies investigated one web-based program each [28,39-56].

The characteristics of the included articles are summarized in Table 1. Studies examined web-based programs delivered in the United States [39-46], Canada [28,47-49], United Kingdom [34,50,51], England [32,33], Sweden [35,52-54], Australia [37,38,55], New Zealand [34,56], Italy [34], and Spain [26,27,36]. Participants included children in their school-aged years (aged 5-18 years): primary school–aged children [26,27,32,33,36,42,50,56], secondary school–aged children [28,34,35,37-41,43-46,51,52,54], or both primary and secondary school–aged children [47-49,53,55]. Children in 24 studies were formally diagnosed or screened to be at higher than normal risk for a health-related condition or risk factor by a health care professional (eg, physicians, allied health practitioners, and nurses) [26-28,32-45,47-49,51-54,56]. One study included families with children who may have self-identified with having a health disorder [55]. Of the included studies, 15 reported the gender of the participating caregivers; mostly mothers participated in the intervention program [32,35,37,39-44,48,50,51,53-55]. A total of 8 studies included information on the parent or caregiver’s academic background, where all caregivers noted completing higher education [32,40,42-44,46,48,49]. Only 2 studies reported the socioeconomic statuses of the families [44,46], where families from both studies mostly reported having an average annual income.

**Table 1:** Study design of included studies and program characteristics.

Study	Study aim	Program characteristics (partly guided or self-guided; frequency or duration of intervention)	Family characteristics	Qualitative data collection method
Guagliano et al [50], 2019; United Kingdom	To assess the feasibility and acceptability of a web-based physical activity intervention (FRESH^a^)	Self-guided; weekly challenges; 6 wk	n=12 families Children^b^: 8-10 y (average 8.3 y) Caregivers^b^: 39.8 y (average) Mostly mothers	Feedback questionnaire and semistructured focus with families at 6 wk
Hatfield et al [37], 2018; Australia	To determine the effectiveness, usability, and barriers and facilitators related to an online transition program for adolescents with autism spectrum disorder program (BOOST-A^c^)	Partly guided; 2 mo	n=39 families Children^b^: secondary school–aged (8-11 y; average 14.8 y); mostly boys Caregivers^b^: mostly mothers; mostly with moderate-high SES^d^	Feedback questionnaire and semistructured interviews with families within 2 mo post program completion
Jogova et al [47], 2013; Canada	To conduct a process evaluation of an online healthy lifestyle program for children with obesity (LiGHT^e^)	Partly guided; 4 wk	n=20 families participated in the intervention Children^f^: 10-17 y (average 13-15.5 y); mostly girls	2-h focus groups with families and feedback questionnaires collected from families post program
Bevan Jones et al [51], 2020; United Kingdom	To evaluate the feasibility, acceptability, and potential impact of an online psychosocial program for children with a history of depression (MoodHwb^g^)	Self-guided; 2 mo	n=44 children; n=31 caregivers Children^b^: ≥13 y (average 16.3 y); mostly girls Caregivers^b^: mostly mothers	Feedback questionnaire and semistructured interviews with families post program completion (2 mo)
Khan et al [32]^h^, 2021; England	To explore the fidelity and experiences of families with a behavioral intervention for children with tics disorder (ORBIT^i^)	Partly guided; 10-12 wk	n=112 families received intervention Children^f^: 9-16 y (average 12y); mostly boys with moderately severe symptoms Caregivers^f^: mostly mothers; completed higher education^j^	Feedback questionnaire and semistructured interviews with families at post program completion (3 mo)
Lalloo et al [39], 2022; United States	To characterize families’ engagement with a pain management intervention for children with SCD^k^ (iCanCope with SCD)	Self-guided	n=56 families; n=1 child received intervention Children^b^: 12-18 y (average 14.8 y); mostly girls Caregivers^b^: mostly mothers	Semistructured interviews with families within 6 mo post program completion
Lenhard et al [52], 2016; Sweden	To describe participants’ experiences with a cognitive behavioral therapy program for children with obsessive compulsive disorder (BiP OCD^l^)	Partly guided	n=21 families received intervention Children^f^: 12-17 y	Interviews with families at 3-6 mo post program completion
Muller et al [55], 2024; Australia	To gain parents’ insights on their engagement with a program for the management of childhood anxiety (BRAVE^m^ Self-Help)	Self-guided	n=14 families received family version Children^b^: 3-17 y Caregivers^f^: average 44y; mostly mothers	Semistructured interviews with caregivers at 6 mo
Murray et al [40], 2022; United States	To evaluate the feasibility and acceptability of a pain management program for children undergoing spinal fusion surgery	Partly guided; weekly or fortnightly modules; presurgery period: 4-6 wk, postsurgery period: 6-8 wk	n=13 families received the intervention Children^b^: 12-17 y (average 14.3 y); mostly girls Caregivers^b^: mostly mothers; mostly completed higher education and with high SES	Semistructured interviews with families 3 mo postsurgery
Nieto et al [36], 2015; Spain	To assess the feasibility of a pain management program for children with functional abdominal pain (DARWeb^n^)	Self-guided; weekly modules	n=15 families received intervention Children^b^: 9-14 y; mostly girls	Feedback questionnaire and semistructured interviews with families at 2 wk post program completion
Nieto et al [26], 2019; Spain	To explore the impact and families’ perspectives of a pain management program for children with functional abdominal pain (DARWeb)	Self-guided; weekly modules	n=22 families Children:f 9-14 y (average 11.23 y); mostly girls and with low pain severity	Semistructured interviews with families at post program completion
Nieto et al [27], 2019; Spain	To evaluate the efficacy of a pain management program for children with functional abdominal pain (DARWeb)	Self-guided; weekly modules	n=25 families received intervention Children^b^: 9-15 y (average 11.28 y); mostly girls	Feedback questionnaire and semistructured interviews with families post program completion (11 wk)
O’Sullivan et al [48], 2018; Canada	To evaluate the acceptability of a self-management program for Irish children with JIA^o^ and their families (TTC^p^)	Self-guided; weekly modules;12 wk	n=20 families received intervention Children^f^: 12-18 y (average 14.19 y); mostly girls Caregivers^f^: mostly 40-49 y; mostly mothers who completed higher education	Focus groups or interviews with families at 2 wk program commencement
Palermo et al [41], 2018; United States	To evaluate the acceptability and feasibility of a cognitive behavioral intervention for children with sickle cell disease and their families (Web-MAP^q^)	Partly guided	n=15 families received CBT^r^ version Children^b^: 11-18 y (average 14.8 y); mostly girls Caregivers^b^: mostly mothers with low to moderate SES	Semistructured with families at post program completion
Sonney et al [42], 2020; United States	To evaluate the feasibility, acceptability and efficacy of a sleep intervention for children with asthma and their families (SKIP^s^)	Self-guided; weekly modules	n=29 families received the intervention Children^b^: 6-11 y (average 8.3 y) Caregivers^b^: mostly mothers who completed higher education	Feedback questionnaire and semistructured interviews with families at 12 wk
Stasiak et al [56], 2018; New Zealand	To evaluate the feasibility and acceptability of a cognitive behavioral intervention for children with mild to moderate anxiety related to the experience of a natural disaster and their families in primary care (BRAVE-ONLINE^t^)	Partly guided; weekly modules	n=42 families received the intervention Children^b^: 7-15 y (average 11.1 y); mostly with generalized anxiety disorder Caregivers^b^: mostly with moderate-high SES	Feedback questionnaires with families at post program completion (12 wk)
Stinson et al [28], 2015; Canada	To explore the usability of a self-management program for children with cancer and their families (Teens Taking Charge: Managing Cancer Online)	Self-guided	n=22 children, n=15 caregivers received intervention Children^b^: 12-18 y (average 15.2 y); mostly boys	Semistructured interviews with families following receipt of intervention
Thompson et al [43], 2019; United States	To evaluate the feasibility and acceptability of a self-management program for children with type 1 diabetes and their families (FTO^u^)	Self-guided; fortnightly modules; 3 mo	n=27 families received intervention Children^f^: 10-15 y; mostly girls Caregivers^f^: mostly mothers who completed higher education and with moderate-high SES	Phone interviews with families post program completion
Thorén et al [53], 2021; Sweden	To explore parents’ experiences with a lifestyle program for children with obesity (Web-COP^v^)	Partly guided; weekly sessions; in-person group sessions–4 wk, web-based modules–12 wk	n=51 families received intervention Children^b^: 5-13 y; mostly girls Caregivers^f^: mostly mothers with history of obesity	Semistructured interviews with caregivers at 2-4 mo post program completion
Wade et al [44], 2017; United States	To assess the feasibility and acceptability of a problem-solving and communication skills training program for children with traumatic brain injury and their families (TOPS^w^)	Partly guided	n=49 families received the family version Children^b^: 11-18 y (average 14.7 y); mostly boys with moderate-severe brain injury Caregivers^b^: mostly mothers who completed higher education and with moderate-high SES	Satisfaction surveys and interviews with families at 6 mo
Wade et al [34], 2021; New Zealand, United Kingdom, and Italy	To adapt a problem-solving and communication skills training program for children with traumatic brain injury and their families (TOPS) in New Zealand, United Kingdom, and Italy	Partly guided; New Zealand: 5 wk	Childrenb: New Zealand: 12-17 y Italy and United Kingdom: adolescent years	Focus groups with caregivers or families following receipt of the intervention in Italy and New Zealand; feedback questionnaire with families in the United Kingdom at post program completion (1 mo)
Yuen et al [45], 2016; United States	To develop and evaluate the usability of a psychoeducation intervention for children affected by a natural disaster and their families (BBN^x^)	Self-guided	n=24 children accessed intervention module Children^f^: 12-17 y (average 14.12 y); mostly girls with some degree of PTSD^y^	Children were observed and provided feedback (verbal and written) while completing selected program module; feedback questionnaire and interviews with children after completing selected program module
Simonsson et al [54], 2021; Sweden	To explore the experiences of families with a treatment program for children with nonsuicidal self-injury disorder and their families (online ERITA^z^)	Self-guided; 12 wk	n=25 families received the intervention Children^f^: 14-17 y; mostly girls Caregivers^f^: 43-55 y; mostly mothers	Semistructured interviews with families post program completion
Lee et al [46], 2023; United States	To evaluate the effectiveness and usability of an intervention on human papillomavirus vaccination for Hmong-American families (Hmong Promoting Vaccines—HmongHPV website)	Partly guided; daily modules; 1 wk	n=30 families received intervention Children^f^: 12-16 y (mostly <15 y) Caregivers^f^: mostly completed higher education and with average SES	Surveys with families post intervention completion (1 and 5 wk); interviews with families at 6 wk post intervention completion
Andersson et al [35], 2024; Sweden	To explore families’ experiences completing an intervention for children with depression (online BA^aa^)	Self-guided; 10 wk	n=11 families received intervention programs Children^f^: 13-17 y (average 15.2 y); mostly boys Caregivers^f^: all mothers	Semistructured interviews with families at post program completion
Connan et al [49], 2019; Canada	To assess the usability of an intervention on the gluten free diet for children with celiac disease and type 1 diabetes and their families	Self-guided	n=20 families recruited Children^f^: mostly secondary school–aged years (average 13.4-13.5 y); mostly girls Caregivers^f^: mostly completed higher education	Families were observed and engaged in usability interviews during and post module completion
Khan et al [33]^h^, 2022; England	To explore the factors influencing the efficacy and engagement of families with a behavioral intervention for children with tics disorder (ORBIT)	Partly guided; 10-12 wk	n=112 families received intervention Childrenb: 9-17 y (average 12.2 y); mostly boys with moderately severe symptoms	Semistructured interviews with families post program completion
Hatfield et al [38], 2017; Australia	To assess the feasibility of an online transition program for adolescents with autism spectrum disorder program (BOOST-A)	Partly guided	n=6 families received intervention Children^f^: secondary school–aged years (10 and 11 y); mostly boys	Surveys with families immediately following completion of each module

^a^FRESH: Families Reporting Every Step to Health.

^b^Baseline characteristics regardless of allocation.

^c^BOOST-A: Better Outcomes and Successful Transitions for Autism.

^d^SES: socioeconomic status; based on the Socio-Economic Indexes for Areas decile or higher SES with an annual income ≥US $70,000.

^e^LiGHT: Living Green, Healthy and Thrifty program.

^f^Characteristics of participants who engaged in the research’s qualitative data collection component.

^g^MoodHwb: *Hwb* translates to hub, lift, or boost in Welsh.

^h^Same study protocol.

^i^ORBIT: Online Remote Behavioural Intervention for Tics.

^j^Higher education refers to completed tertiary education.

^k^SCD: sickle cell disease.

^l^BiP OCD: BarnInternetProjektet obsessive-compulsive disorder.

^m^BRAVE: Body signs, Relax, Activate helpful thoughts, Victory over fears, Enjoy yourself.

^n^DARWeb: Dolor Abdominal Recurrente web-based intervention.

^o^JIA: juvenile idiopathic arthritis.

^p^TTC: Teens Taking Charge.

^q^Web-MAP: Web-based Management of Adolescent Pain.

^r^CBT: cognitive behavioral therapy.

^s^SKIP: Sleep Intervention for Kids and Parents.

^t^BRAVE-ONLINE: Body signs, Relax, Activate helpful thoughts, Victory over fears, Enjoy yourself - online program.

^u^FTO: Family Teamwork Online.

^v^Web-COP: Web-based childhood obesity prevention.

^w^TOPS: Teen Online Problem Solving.

^x^BBN: Bounce Back Now.

^y^PTSD: posttraumatic stress disorder.

^z^ERITA: Emotion Regulation Individual Therapy for Adolescents.

^aa^BA: Behavioural Activation.

Most of the web-based programs were developed for the management of a medical condition (24 distinct interventions for the treatment or management of 17 unique health-related conditions: autism [37,38], overweight or obesity [47,53], depression [35,51], tic disorder [32,33], sickle cell disease [39,41], obsessive compulsive disorder [52], anxiety [55,56], spinal fusion [40], functional abdominal pain [26,27,36], juvenile idiopathic arthritis [48], sleep disturbance related to having asthma [42], cancer [28], type 1 diabetes [43,49], celiac disease [49], traumatic brain injury [34,44], nonsuicidal self-injury [54], and posttraumatic stress disorder [45]). Two interventions were developed to influence health-related behaviors in school-aged children (unrestricted to a medical diagnosis or condition) [46,50]. All but 2 interventions were entirely technology based (no in-person elements) [37,53]. A total of 19 studies reported on the intervention program’s frequency [26,27,34,36,40,42,43,46,48,50,53,56] or length [32,33,35,37,39,40,43,46-48,50,51,53,54], where interventions mostly included weekly modules and lasted 4 to 12 weeks.

A total of 13 interventions were self-guided [26-28,35,36,39, 42,43,45,48-51,54,55], of which 7 interventions included other eHealth components [26,27,36,39,42,43,50,54,55]. The eHealth components had personalized functions in 3 programs (ie, pedometer [50] and a mobile app used for monitoring or tracking purposes [39,54]) and automated functions in 4 programs (ie, email reminders [26,27,36,42,43,55]). Studies also examined partly guided programs [32-35,37,40,41,44,46,47,52,53,56]. A total of 12 interventions included adjunctive support from professionals (eg, doctoral or postdoctoral research fellows, psychologists, and exercise specialists) who used eHealth technologies such as a videoconferencing platform [34,44], a phone [32,33,35,40,47,56], an email [47], and a built-in messaging platform [32,33,35,52,56]. Adjunct features of included programs are summarized in Table 2.

**Table 2:** Key features of the intervention program (family-based version) of included studies.

Study		Purpose of intervention program	Key features of the intervention program															
			Email^a^		Phone^b^		Videoconf^c^		Built-in messaging platform		Smartphone app		Other eHealth monitoring technology^d^		In-person sessions		Support from a health care professional or expert	
Guagliano et al [50], 2019	To increase physical activity among families													✓ (pedometer)				
Hatfield et al [37], 2018; [38], 2017	To support children with autism to prepare for life outside of school															—^e^		✓(champions [var^f^])
Jogova et al [47], 2013	To support children with obesity to build a healthy lifestyle behaviors or habits			✓ (resp^g^)		✓ (resp)												✓ (exercise specialist)
Bevan Jones et al [51], 2020	Psychosocial program to support children with a history of depression																	
Khan et al [32], 2021; [33], 2022	To support symptom management for children with tics disorder											✓ (stopwatch)						✓ (therapist)
Lalloo et al [39], 2022	To support symptom management for children with sickle cell disease											✓						
Lenhard et al [52], 2016	Treatment program for children with obsessive compulsive disorder									✓								✓ (psychologist)
Muller et al [55], 2024	Treatment program for children with anxiety			✓ (rem^h^)														
Murray et al [40], 2022	To support symptom management for children undergoing spinal fusion surgery					✓ (call)												✓ (postdoctoral research fellows in psych^i^)
Nieto et al [36], 2015; [26,27], 2019	To support pain management for children with functional abdominal pain			✓ (rem)														
O’Sullivan et al [48], 2018	To support symptom management for Irish children with juvenile idiopathic arthritis																	
Palermo et al [41], 2018	To support symptom management for children with sickle cell disease									✓								✓ (therapist; MS^j^ level or postdoctoral research fellows in psychology)
Sonney et al [42], 2020	To improve sleep in children with asthma			✓ (rem)														
Stasiak et al [56], 2018	Treatment program for children with mild to moderate anxiety related to the experience of a natural disaster					✓ (call)				✓								✓ (therapist)
Stinson et al [28], 2015	To support the management of symptoms for children with cancer																	
Thompson et al [43], 2019	To support the management of type 1 diabetes in children			✓ (rem)														
Thorén et al [53], 2021	To support lifestyle changes in children with obesity															✓		
Wade et al [44], 2017	To enhance problem-solving and communication skills in children with traumatic brain injury							✓										✓ (therapist; psychologist or graduate student)
Wade et al [34], 2021	To enhance problem-solving and communication skills in children with traumatic brain injury in New Zealand, United Kingdom, or Italy							✓ (Italy and United Kingdom)										✓ (therapist)
Yuen et al [45], 2016	Treatment program for children affected by a natural disaster																	
Simonsson et al [54], 2021	Treatment program for children with nonsuicidal self-injury disorder											✓						
Lee et al [46], 2023	To improve the human papillomavirus vaccine rates among Hmong-American families					✓ (rem)								✓ (GPS locator)				✓ (Hmong-American health navigator)
Andersson et al [35], 2024	Treatment program for children with depression					✓ (therapist-supported version–phone calls)				✓ (therapist-supported version)								✓ (therapist-supported version—clinical psychologist)
Connan et al [49], 2019	Education program on the gluten free diet for children with coeliac disease and type 1 diabetes and their families																	

^a^Reminder (automatic) or responsive communication.

^b^Reminder (responsive communication) or session call.

^c^Videoconf: videoconferencing.

^d^For example, pedometer, stopwatch, or GPS locator.

^e^Cannot tell.

^f^var: variable.

^g^resp: responsive.

^h^rem: reminder.

^i^psych: psychology.

^j^MS: master’s.

Qualitative data were mostly collected using semistructured interviews conducted at post program completion (3 months post program completion or shorter) [26-28,32,33,35-37,39-46, 48,50-55]. A total of 23 studies examined the experiences of the family unit [26-28,32-37,39-44,46-52,54-56], with only 1 study focusing solely on the point of views of the participating caregivers [53], and 1 study reporting on feedbacks of the participating children [45]. All except 1 study involved the delivery of the web-based program in its entirety to families; 1 study explored families’ experiences with the intervention following their completion of one representative program module [45].

### Quality Appraisal

The Critical Appraisal Skills Programme checklist [29] completed for each study is summarized in Table 3. All studies were clear about their research aims, included qualitative methodology appropriately, and discussed the value of the research. All except 1 study [34] clearly described the recruitment strategy or the collection of all data. Similarly, all except 2 studies [38,44] clearly described the research design. All studies considered ethical issues; however, 1 study was unclear whether ethics approval was obtained [45]. Most studies adequately described the analysis process [26,27,35-37, 40,42,45-48,51-55]. Most studies also provided a clear statement of findings [26-28,33,35-37,40-43,45,47-49,51-55]. It was unclear whether most studies adequately considered the relationship between researcher and participants; most studies lacked reports of authors’ reflexivity or information on whether the researcher critically examined their own role, potential bias and influence during study design, data collection, analysis, and presentation [26-28,32-34,36,38-51,53,54,56].

**Table 3:** The Critical Appraisal Skills Programme qualitative studies checklist.

Study	Aim	Method	Design	Recruitment	Data collection	Relationship	Ethical	Data analysis	Findings	Value
Guagliano et al [50], 2019	Yes	Yes	Yes	Yes	Yes	No	Yes	Uncertain	Uncertain	Yes
Hatfield et al [37], 2018	Yes	Yes	Yes	Yes	Yes	Yes	Yes	Yes	Yes	Yes
Jogova et al [47], 2013	Yes	Yes	Yes	Yes	Yes	Uncertain	Yes	Yes	Yes	Yes
Bevan Jones et al [51], 2020	Yes	Yes	Yes	Yes	Yes	Uncertain	Yes	Yes	Yes	Yes
Khan et al [32], 2021	Yes	Yes	Yes	Yes	Yes	Uncertain	Yes	Uncertain	Uncertain	Yes
Lalloo et al [39], 2022	Yes	Yes	Yes	Yes	Yes	No	Yes	Uncertain	Uncertain	Yes
Lenhard et al [52], 2016	Yes	Yes	Yes	Yes	Yes	Yes	Yes	Yes	Yes	Yes
Muller et al [55], 2024	Yes	Yes	Yes	Yes	Yes	Yes	Yes	Yes	Yes	Yes
Murray et al [40], 2022	Yes	Yes	Yes	Yes	Yes	No	Yes	Yes	Yes	Yes
Nieto et al [36], 2015	Yes	Yes	Yes	Yes	Yes	No	Yes	Yes	Yes	Yes
Nieto et al [26], 2019	Yes	Yes	Yes	Yes	Yes	No	Yes	Yes	Yes	Yes
Nieto et al [27], 2019	Yes	Yes	Yes	Yes	Yes	No	Yes	Yes	Yes	Yes
O’Sullivan et al [48], 2018	Yes	Yes	Yes	Yes	Yes	No	Yes	Yes	Yes	Yes
Palermo et al [41], 2018	Yes	Yes	Yes	Yes	Yes	No	Yes	No	Yes	Yes
Sonney et al [42], 2020	Yes	Yes	Yes	Yes	Yes	No	Yes	Yes	Yes	Yes
Stasiak et al [56], 2018	Yes	Yes	Yes	Yes	Yes	No	Yes	No	Uncertain	Yes
Stinson et al [28], 2015	Yes	Yes	Yes	Yes	Yes	No	Yes	Uncertain	Yes	Yes
Thompson et al [43], 2019	Yes	Yes	Yes	Yes	Yes	No	Yes	Uncertain	Yes	Yes
Thorén et al [53], 2021	Yes	Yes	Yes	Yes	Yes	Uncertain	Yes	Yes	Yes	Yes
Wade et al [34], 2021	Yes	Yes	Yes	Uncertain	Yes	No	Yes	Uncertain	Uncertain	Yes
Wade et al [44], 2017	Yes	Yes	Uncertain	Yes	Uncertain	No	Yes	No	Uncertain	Yes
Yuen et al [45], 2016	Yes	Yes	Yes	Yes	Yes	No	Uncertain	Yes	Yes	Yes
Simonsson et al [54], 2021	Yes	Yes	Yes	Yes	Yes	Uncertain	Yes	Yes	Yes	Yes
Lee et al [46], 2023	Yes	Yes	Yes	Yes	Yes	Uncertain	Yes	Yes	Uncertain	Yes
Andersson et al [35], 2024	Yes	Yes	Yes	Yes	Yes	Yes	Yes	Yes	Yes	Yes
Connan et al [49], 2019	Yes	Yes	Yes	Yes	Yes	Uncertain	Yes	Uncertain	Yes	Yes
Khan et al [33], 2022	Yes	Yes	Yes	Yes	Yes	No	Yes	Uncertain	Yes	Yes
Hatfield et al [38], 2017	Yes	Yes	Uncertain	Yes	Yes	No	Yes	Uncertain	Uncertain	Yes

### Results of Data Syntheses

The key themes identified on families’ experiences with family-focused web-based health programs were (1) connecting with others, (2) agency of learning, (3) program reputability or credibility, (4) program flexibility, (5) meeting participants’ needs regarding program content or delivery, and (6) impact on lifestyle. Themes reported by authors of included studies are summarized in Multimedia Appendix 3 [26-28,32-56].

#### Theme 1: Connecting With Others

Connecting with others related to forming quality relationships and established by the constructs of the web-based program and the influence of interpersonal relationships on families’ experiences with the intervention. Building new or strengthening existing relationships encouraged the uptake of and engagement with treatment [32,36,39,42,43,50,52-55]. Subthemes included connecting with own family members (internal), other families (external), experts or health care professionals, and artificial intelligence (AI; eg, relational or conversational agent and chatbot).

##### Connecting With Own Family Members (Internal)

Families reflected on connecting with own family members during the intervention. The interventions created an opportunity for family members to involve themselves or realize their roles in making changes to support health collectively [28,35,37,39,42,43,47,50,52-54] or complete the intervention [27,32,33,36,39,43,47,50,52,53]. Families described the active participation of the entire (ie, immediate) family or at least one other family member (eg, sibling, other parent, or grandparents) in the program, including having “within-family competitions” [50] and nonparticipating siblings using program resources [53]. Parents and children expressed the importance of involving the family unit or parents to achieve goals or engage in the web-based program [27,32,35,36,39,42,43,50,52-54]. For instance, parents were key supporters of participating children and motivated program engagement [27,32,35,36,39,42,50, 52,53]. Some parents reported that their motivation to engage with the program was dependent on their child’s engagement [55]. Children also positively perceived their parent’s involvement throughout the program; children felt less alone and found it beneficial to have their parent partake with them [35,52].

Families described improved relationships with each other post program completion [35,44,50,54]. Parents suggested that the program helped them better understand their child and their experiences [35,54], “opened lines of communication” [47] or allowed them to “have common language” [54] that was not possible before the program. Similarly, parents wanted the intervention to provide more structured opportunities for them to converse or complete an activity with their child [46,54].

##### Connecting With Other Families (External)

Families expressed the benefits of having the opportunity to connect with other families during the intervention [36,39,47,48,50,53,55]. Parents recognized that establishing a sense of community or network was an important feature of conventional treatment programs and recommended these elements to be preserved in eHealth interventions [47,55]. Families suggested that building relationships and interacting with other families, whether through group sessions or eHealth technologies or program features, supports their engagement [53,55] with and enjoyment [39] of the program. Parents indicated that having the opportunity to connect with other families throughout the program was reassuring for them and helped them know that they are “not alone with [their] struggles” [55]. Families suggested that eHealth intervention features, including shared forums, web-based chats, and support groups, enable the exchange of ideas [36,39,47,53] and shared experiences [47,48,53,55].

##### Connecting With Experts, Health Care Professionals, or Responsive AI

Families conveyed the importance of receiving support from professionals [32,35,37,44,47,52-56] or AI [43]. Families appreciated the responsive support received from health care professionals or AI to navigate the treatment [37,55], address queries [33,35,43,47,52-54], or maintain their confidence or motivation throughout the program [32,35,52,55]. Families described connecting with or feeling supported by a health care professional—actual human [32,52]—or AI [43], like they “had never been before.” For instance, families who participated in partly guided web-based programs, reflected on the increased accessibility and strengthened connections established with experts, including increased “self-disclosure” [52] and the likelihood of families to “ask direct questions at any time” [53] or any questions freely [54]. Some children noted that program materials may be used to enhance sessions with experts in conventional services (ie, during hybrid care) or serve as an alternate intervention in between sessions [51].

Families also expressed that having interactions and support from human experts preserved the merit of conventional services [54], including the notion that professionals “wanted to help them” [54] and were on their side [52]. Participants noted favorable features of these relationships (both human-to-human or human-to-AI): the responders being prompt [35,43,47,52,54], optimistic [47], and personable [35,43,52,54]. Conversely, some families suggested the need for in-person contact with experts to establish or re-establish engagement with treatment [52,55]. Having contact with an actual person was preferred by some families over interacting with AI [43,52,54]. Families from the study on a self-guided web-based program with inbuilt support from a relational agent noted the limited ability of AI to meet unique needs [43]. Similarly, families who completed partly guided intervention programs noted their ambivalence with having all things digital or receiving responses from “robots” (automated response) [52,54].

#### Theme 2: Agency of Learning

Agency of learning related to an individual’s sense of self-responsibility for their learning and includes their intention, self-efficacy, autonomy, and motivation. The increase in learning agency noticed by participating children or caregivers in themselves or others influenced program uptake, engagement, or sustainability [32,35,37,39,42,47,50-54]. Families often reported that the participating children had low agency, namely low self-confidence or motivation for or ownership of plans to improve their health in the beginning of the intervention [37,39,47,50,53,55]. Parents also conveyed similar reluctance and low motivation in their family to engage with program activities, related to having negative preconceived thoughts (eg, having apprehension about completing an activity well) [37,50]. Conversely, some families perceived that the structure or contents of the intervention aligned with their intent and respected their autonomy, which supported their engagement and positive experiences [52,54,55]. Some children enjoyed “feeling that [they] had the treatment to [themselves] and [helped themselves] rather than just [receiving] help” [54]. Families described that the program provided resources, such as new knowledge and ideas, that empowered them to take active roles to improve their health; they gained an understanding [26,27,46,53,54] of and ways to address [37,39,42,46,51-54] their symptoms, condition, or situation. Families also reflected that goal setting was facilitated by the intervention and strengthened learner agency [39,40,42,47,51]; goal setting helped families recognize that change was “manageable and possible” [47]. They suggested achieving goals or making progress further empowered them [42,47,51].

However, some children recounted that increased self-responsibility influenced negative experiences throughout the intervention [54,55]. Whereas children appreciated the independence to manage and have ownership of their treatment or changes, their perceived failure to achieve goals and complete activities increased feelings of “shame and guilt and decreased self-confidence” [54]. Families also expressed that the self-guided nature of the intervention (ie, intervention encouraging independence) was counterproductive when they had “doubts about their own capabilities” [54] or “whether they were completing the program correctly [or] on the right track” [55]; some families experienced moments of decreased self-confidence during the program [54,55]. Some children expressed that these negative experiences reminded them of their disorder and decreased agency [54]. When participants had low learning agency, they were poorly engaged with the intervention [26,50,53,55].

#### Theme 3: Program Reputability or Credibility

Knowledge of the program developers or affiliates impacted families’ engagement with the intervention. For instance, families perceived programs developed by well-known institutions or health care professionals as trustworthy and were therefore motivated to uptake or engage with the web-based program [47,55]. Similarly, families noted being more likely to participate in and trust programs recommended to them by “friends, allied health professionals, hospitals and educational facilities” [55]. Families expressed the desire to learn from these sources; participating in the program was their opportunity to access the evidence-based knowledge [55]. Families also regarded the intervention as credible when the information presented matched their prior knowledge or lived experiences [28].

#### Theme 4: Program Flexibility

Program flexibility relates to the adaptability of program components into families’ ways of living and family’s reflections on the ease of program use. Families expressed that they were able to adapt the intervention to their lifestyles [27,35,36,39,43-45,47,50,52-54]. For instance, families noted that they were able to choose when to engage with the program or at their own pace, including using program resources and scheduling services when deemed most necessary, while adhering to the overall timeline of the web-based program [27,35,36,47,52-54,56]. Engaging in the program also required an acceptable amount of their time [39,43,47] or was a valuable use of their time [42,47]. Families reported that they preferred the web-based delivery of the intervention [27,35,36,52,56]; the program supported their ability to “prioritize other activities in life, as well as [fit] the treatment into [their] weekly schedule” [52]. Families suggested that program features, such as the compatibility of the intervention across multiple devices [40,55] and being able to freely navigate content (eg, return to completed sections or skipping content) [46,49,55] were favorable and supported program engagement.

On the other hand, some families reflected on difficulty participating in the intervention due to a lack of time [26,35,39,50,53,55], competing priorities [26,32,34,35,39,50, 55], or a lack of energy from a demanding schedule [39]. Families also described the incompatibility of the intervention program to their preferred platform as an inconvenience, which further “wasted” time (eg, families mentioned website features that did not work on their preferred digital device) [32,36-38,51,56].

#### Theme 5: Meeting Participants’ Needs Regarding Program Content or Delivery

Families reflected on whether the intervention met their needs regarding the intervention program’s content or delivery, including the appropriateness of program content for children or a subset of children with respect to their age or disease and symptom severity. Families expressed that positive experiences with the program were related to learning new, tangible information (eg, ideas and knowledge) [26,27,33,35-37,39-44, 46-49,52-56]. Before participating in the intervention program, families shared that most of their condition-specific knowledge were retrieved from searching the internet [49]. Families conveyed that including condition-specific elements [28,36,37,39-41,43,44,48,51,52] and age-appropriate language or content [39,47,51] supported program engagement. Families suggested that program design features often perceived to support children’s participation or understanding of the content included, having greater use of friendly and complementary visuals [27,28,41,42,45,47,49,51,56], videos or animations [33,37-39,42,47], interactive content particularly for younger primary school–aged children [28,33,39,40,42,46,47,49,53], simple wording [28,35,39,47,51], affirmative or respectful language [51,53], and customizable features [28,34,39,45-48, 51], having rewards particularly physical rewards [33,50, 51], and being easy to navigate [28,39,41,42,45,49,51,53,55,56]. Families described that relatable and personable features of the program, such as the use of storylines featuring children as the main characters or case studies portraying relatable scenarios further motivated or encouraged their participation [27,28,34,36, 40,43-45,47,51,52,54,55].

Parents suggested that program elements that were unappealing to children included having an excessive amount of text [27,36,47] or using advanced language or jargon [47]. Families described the intervention as unsupportive of their needs when they found the program’s content redundant [27,32,39,42,47], too simple [47], lengthy [38,39,49], or irrelevant [35,39,55]. Families also wanted the intervention to include further or more specific information on their condition, health-related behaviors, or skills [35,37,43,49,51]. Families found the program irrelevant when they had prior education or lived experiences with the disorder or situation for a longer duration [35,55]. Such families often perceived the intervention as an “immediate lifeline” [55] or early intervention for participants who were first diagnosed with or having an early onset of a condition or experiencing mild symptoms [28,39,48,51,55]. Conversely, some families perceived the intervention being more suitable for children experiencing more severe symptoms [26,35]. Families recommended the development of different versions of the program to meet the needs of children in different age groups; children in older age groups found the program too simple and unengaging [32,33,47].

#### Theme 6: Impact on Lifestyle

Families reflected on the sustained health-related behavioral changes they noticed or actively made during or following program completion, which impacted their lifestyle. Families described that the intervention was a first step that helped them improve engagement in health-supporting behaviors or establish health-supporting routines, including healthy eating, physical activity, and sleep [40,42,50,53]. For instance, parents noted that because their participation in the intervention program, they set new boundaries at home to support healthy behaviors or habits [53]. Some families reported that they continued practicing techniques or skills learned from the program, which in turn enhanced their quality of life [27,35,36,40,54] or allowed them to “return to regular activities” [35,40], even “normal life at 100%” [27].

## Discussion

### Principal Findings

This review identified 28 articles on 26 studies, resulting in 6 themes describing children or caregivers’ perception of family- and web-based eHealth interventions. Descriptions of each theme encompassed families’ reflections on program features regarded as valuable to them and that influenced their uptake of or engagement with the intervention.

### Comparison With Existing Literature

On the basis of the studies included in this review, families mostly had positive experiences using web-based programs supporting health. This review found that families positively perceived elements of the web-based program that imitated features of their conventional counterparts delivered in person. Existing literature on eHealth interventions for children and their families suggests that as with essential features of conventional interventions, eHealth interventions should incorporate behavioral or self-management principles involving the family unit, such as symptom management, lifestyle behaviors, relationship management, and psychosocial management [3,12]. Similarly, this review found that families favored self-guided features aligned with behavioral and self-management principles of the web-based programs, including goal setting; self-monitoring and self-reflection tasks; having interactive, tangible, relatable, and credible content; and personable activities that are easily adaptable to daily living (ie, themes: agency of learning, program reputability or credibility, program flexibility, and meeting participants’ needs regarding program content or delivery). The web-based nature of web-based eHealth programs further empowers families during treatment by enabling flexibility to access resources when needed and unconstrained by locality and in whether or how resources (eg, information) are used [57]. Families also shared that such features enabling this flexibility encouraged their engagement with the intervention or health-supporting behaviors.

The included studies highlighted the importance of quality relationships to bolster the health and well-being of children and their families. Evidence suggests that children with chronic health conditions feel lonelier compared to children lacking health conditions [58]. Loneliness detrimentally impacts health, including the trajectory of health conditions, and hinders children from seeking help or communicating with others [58]. The proliferated use of technology affects social interactions, and passive use (ie, using technology in a way that decreases opportunities for social connectedness) also risks exacerbated feelings of loneliness [59]. This review found that web-based eHealth interventions can promote connections for families—including families with children with health conditions—within and outside the family unit (ie, theme: connecting with others). As suggested by other literature examining children’s perspectives of digital technologies, opportunities to engage with web-based communications preserved or enhanced children’s relationships with others (eg, family members and friends) [57]. Findings from this review suggest that web-based eHealth programs can cultivate quality connections with others, regardless of the absence of in-person interactions, and may therefore influence health-related outcomes in children. Our findings extend existing evidence on the health-supporting effects of social connections by providing insight into the benefits of designing web-based programs involving the family unit (ie, subtheme: connecting with own family members). A web-based program that involves children and their families, or at least a caregiver, can satisfy the sense of relatedness required for children to engage in the desired health-promoting activities or behaviors. Existing literature recognizes the importance of the social environment and personable support to facilitate learning and influence children’s self-management of or responsibility for their health [3,60-62]. Web-based program features that enable social interactions encourage active learning; individuals are more likely to apply learned behaviors when informational and emotional support are received from others [60-62]. Children, in particular, learn through modeling or observing others [61,62]. School-aged children, particularly primary school–aged children (ie, children <12 years of age), significantly depend on their caregivers to manage their health; caregiver involvement is commonly characteristic of conventional behavioral interventions for children [8]. Depending on the level of guidance provided by the web-based program, direct digital translations of conventional health care services may lack humanized elements, including psychosocial support or human connections, which may deter achieving intended health outcomes [63]. We also found that families valued human connections and were more likely to engage with the intervention when the program supported or enhanced their relationships with others, including children with their caregivers and vice versa, families with other families with children undergoing similar health conditions or situations, and health care professionals (ie, theme: connecting with others).

Novel insights were gained on families’ relationships with health care professionals or the role of health care professionals in family-focused web-based programs (ie, subtheme: connecting with experts, health care professionals, or responsive AI). It was found that families improved their communication with a health care professional throughout the web-based program. Participating children may have found comfort with the web-based nature of the intervention—related to them being able to participate in the intervention at a physical environment of their choice—which supported them to feel safe and actively communicate or build a rapport with others [57,61]. Web-based programs can cultivate collaborative relationships or social engagements between participating families and involved health care professionals, supported by features including web-based discussion forums, inbuilt messaging platforms, and phone or videoconferencing platforms. This collaborative relationship further increases the likelihood that children engage with the intervention (ie, the learning process) and assume increased responsibility for their health [60,61].

This review found that families may prefer support received from human-to-human over human-to-AI interactions throughout the intervention (ie, subtheme: connecting with experts, health care professionals, or responsive AI). This is consistent with previous literature on the limitations of AI in eHealth interventions to support the specialized or therapeutic needs of patients [63,64]. However, this review also noted families’ appreciation for responsive AI when included in web-based programs due to its rapid responses and accessibility. Generative and responsive AI is rapidly developing, with increased efforts to humanize AI features and evidence that AI technology has the potential to provide compassionate health care [65,66]. It is important to note that the limitations of AI found in this review may not be applicable in the future [65]. Involving families in the development of AI features in eHealth interventions is essential for ensuring acceptable family-centered education [67].

This review also identified unmet needs experienced by families with the web-based program (ie, theme: meeting participants’ needs regarding program content or delivery). Families noted unsatisfied informational needs, where the content was described as being too simple or irrelevant to them, given their age, education background, or experience with the pediatric health condition. Children have individual and dynamic needs with age and stage or degree of health conditions [8,60,64]. This finding suggests that web-based programs may satisfy the foundational needs of children and their families when managing health conditions. However, more complex and specialized needs, including diminishing motivation or troubleshooting decreased self-confidence or self-management experienced during eHealth interventions, may require support from a health care professional or more sophisticated integration of contemporary AI [12,64]. On the other hand, this finding may also suggest that web-based eHealth interventions can create opportunities for families to realize their unmet health-related needs or intentions to seek further support from health care professionals [57].

### Strengths and Limitations

This is the first qualitative systematic review, to the researchers’ knowledge, that synthesizes research reported in the last decade on the experiences of both the participating children and caregivers with family-focused web-based intervention programs. Whereas this review applied a search strategy that involved Boolean searching versus, for example, subject searching and was translated across databases to identify articles that met the eligibility criteria, the evidence base on family-focused web-based programs to improve children’s health is still developing. The search method of this review was therefore chosen to maximize flexibility. The search strategy was also confirmed with a university librarian to ensure focus. A limitation of the identified studies is the lack of clarity on whether researchers engaged in reflexivity, which is a core component to ensure transparency of the research process and quality of qualitative research. Limitations of this review also include that findings are representative of research from economically advanced countries, where resources, including access to the internet, may be more available. It is unclear whether this review equitably captures the perspectives of families from diverse populations, as not all studies have reported on characteristics that were found to influence participants’ experiences with eHealth interventions, including caregivers’ education background or socioeconomic information [64,65]. Similarly, families in the identified studies may have also disproportionally conveyed positive feedback with the web-based programs. This review also explored the experiences of the family unit. Most studies in this review reported on the combined perspectives of participating children and their caregivers. Children, caregivers, and other family members may have different understandings of or priorities and motivators with children’s health that may influence their experiences with treatment or health management, including eHealth interventions. More research is needed to distinguish these perspectives of children, caregivers, caregiver-child dyads, and other family members on family-focused eHealth interventions and across disease trajectories and children’s developmental stages. This review focused on web-based programs directed at the family unit, which may exclude families’ experiences with other types of family-focused eHealth interventions such as smartphone app–based programs. This review also presents a snapshot of findings on families’ experiences with web-based eHealth programs in the last decade. With progressive advancements in digital technologies and AI and families’ adaptations to the digital culture, continued exploration of this phenomenon is needed to inform the development of family-focused eHealth interventions.

### Implications for Practice

On the basis of the findings of this review, key considerations when developing eHealth interventions for improving children’s health and incorporating eHealth in health care systems include those outlined in Textbox 1.

**Key considerations for developing eHealth interventions to improve children’s health.**

- Children and their families highly regard eHealth interventions directed at the family unit and that create opportunities for cultivating quality social connections within or external to participating families. Web-based education may be supported by interventions that leverage features recognized by families to influence children’s agency or self-management skills relative to improving their health: goal setting, self-monitoring and self-reflection tasks, interactive, tangible, relatable and evidence-based content, and personable activities that are easily adaptable to daily living.
- Family members, namely caregivers, were identified as social factors to support children’s engagement in health-supporting activities and behaviors. Self-guided eHealth interventions require features that are positively perceived by families and related to foundational or informational needs of families lacking prior experiences with the health condition and treatment.
- Families highlighted that web-based programs need to be adaptable to meet children’s changing needs throughout the program, including features that respond to fluctuations in motivation and their often-dynamic experience of their health conditions.
- eHealth (ie, web-based) interventions may present opportunities for families to recognize unmet health-related needs.
- Responsive features have the potential to humanize self-guided eHealth interventions. Advancements in responsive AI may serve similar functions as health care professionals in web-based programs, including meeting families’ health-related needs.

### Conclusions

This review synthesized the evidence on families’ experiences with family-focused eHealth (ie, web-based) interventions. Insights were gained on the potential of eHealth interventions to satisfy health-related needs of children and their families. Key considerations presented in this review highlight the need for program developers and health care systems to adapt the elements of eHealth interventions to child developmental stages and the complex dynamism of childhood health conditions. In doing so, the benefits of eHealth interventions may be maximized to meet the growing number of families needing treatment for childhood health conditions. More research is needed to equitably tailor family-focused eHealth (ie, web-based) interventions to diverse populations and explore this phenomenon in the context of novel advancements in digital technologies and AI. Future research could also distinguish the perspectives of children, their caregivers, and other family members on eHealth interventions. These considerations can be used to enhance the design of family-focused eHealth interventions and better inform their inclusion in treatment plans to improve children’s health.

## Data Availability

The datasets generated and analyzed during this study are available from the corresponding author on reasonable request.

## Appendix

Multimedia Appendix 1 PRISMA (Preferred Reporting Items for Systematic Reviews and Meta-Analyses) checklist.

Multimedia Appendix 2 Search strategy per database.

Multimedia Appendix 3 Themes or subthemes, categories, or codes reported in the results section of included studies.

## Abbreviations

AI: artificial intelligence
PRISMA: Preferred Reporting Items for Systematic Reviews and Meta-Analyses
