# Supplementary material for: Families’ Experiences With Family-Focused Web-Based Interventions for Improving Health: Qualitative Systematic Literature Review
Source: J Med Internet Res. 2025 Jan 30;27:e58774. doi: 10.2196/58774 (PMC11826954; doi:10.2196/58774)
Supplement: Multimedia Appendix 2 [file jmir_v27i1e58774_app2.docx]

## Multimedia Appendix 2 – Search strategy per database

Medline

| # | Key words or MeSH terms |
| --- | --- |
| Population | |
| 1 | famil*.ti,ab |
| 2 | child*.ti,ab |
| 3 | parent*.ti,ab |
| 4 | Child rearing/ |
| 7 | parent-child relations/ |
| 8 | father-child relations/ |
| 9 | mother-child relations/ |
| A: 1 OR 2 OR 3 OR 4 OR 7 OR 8 OR 9 | |
| Intervention | |
| 11 | eHealth.ti,ab |
| 12 | e-Health.ti,ab |
| 13 | e-therapy.ti,ab |
| 14 | e-intervention.ti,ab |
| 15 | ((online or digital or web* or internet* or electronic* or self-administered) adj3 (health or therap* or intervention* or program)).ti,ab |
| 20 | Internet/ |
| 22 | Digital Technology/ |
| 23 | Internet-based intervention/ |
| B: 11 OR 12 OR 13 OR 14 OR 15 OR 20 OR 22 OR 23 | |
| Outcome | |
| 25 | experienc*.ti,ab |
| 26 | accept*.ti,ab |
| 27 | satisfaction.ti,ab |
| 28 | perspective*.ti,ab |
| 29 | usability.ti,ab |
| 30 | perception*.ti,ab |
| 31 | attitude*.ti,ab |
| 33 | exp "patient acceptance of health care" |
| 34 | exp patient satisfaction/ |
| C: 25 OR 26 OR 27 OR 28 OR 29 OR 30 OR 31 OR 33 OR 34 | |
| Study | |
| 36 | interview*.ti,ab |
| 37 | Qualitative*.ti,ab |
| 38 | focus group*.ti,ab |
| 39 | mixed method*.ti,ab |
| 40 | feasibility.ti,ab |
| 41 | interview/ |
| 42 | exp qualitative research/ |
| 43 | focus groups/ |
| 44 | feasibility studies/ |
| D: 36 OR 37 OR 38 OR 39 OR 40 OR 41 OR 42 OR 43 OR 44 | |
| E: A AND B AND C AND D | |
| English language and year (2012) limits applied to E | |

Cochrane Library

| # | Key words or MeSH terms |
| --- | --- |
| Population | |
| 1 | famil*:ti,ab |
| 2 | child*:ti,ab |
| 3 | parent*:ti,ab |
| 6 | [child rearing] this term only |
| 7 | [parent-child relations] explode all trees |
| A: 1 OR 2 OR 3 OR 4 OR 5 OR 6 OR 7 | |
| Intervention | |
| 11 | eHealth:ti,ab |
| 12 | e-Health:ti,ab |
| 13 | e-therapy:ti,ab |
| 14 | e-intervention:ti,ab |
| 19 | ((online or digital or web* or internet* or electronic* or self-administered) NEAR/3 (health or therap* or intervention* or program)):ti,ab |
| 20 | [internet] this term only |
| 22 | [digital technology] explode all trees |
| 23 | [internet-based intervention] explode all trees |
| B: 11 OR 12 OR 13 OR 14 OR 19 OR 20 OR 22 OR 23 | |
| Outcome | |
| 25 | experienc*:ti,ab |
| 26 | accept*:ti,ab |
| 27 | satisfaction:ti,ab |
| 28 | perspective*:ti,ab |
| 29 | usability:ti,ab |
| 30 | perception*:ti,ab |
| 31 | attitude*:ti,ab |
| 33 | [patient acceptance of health care] explode all trees |
| 34 | [patient satisfaction] explode all trees |
| C: 25 OR 26 OR 27 OR 28 OR 29 OR 30 OR 31 OR 33 OR 34 | |
| Study | |
| 36 | interview*:ti,ab |
| 37 | Qualitative*:ti,ab |
| 38 | focus group*:ti,ab |
| 39 | mixed method*:ti,ab |
| 40 | feasibility:ti,ab |
| 41 | [qualitative research] explode all trees |
| 42 | [focus groups] explode all trees |
| 43 | [feasibility studies] this term only |
| D: 36 OR 37 OR 38 OR 39 OR 40 OR 41 OR 42 OR 43 | |
| E: A AND B AND C AND D | |
| English language limit applied to E & Date published on the Cochrane library between Oct 2012 and present | |

Embase

| # | Key words or EMTREE terms |
| --- | --- |
| Population | |
| 1 | famil*.ti,ab |
| 2 | child*.ti,ab |
| 3 | parent*.ti,ab |
| 4 | family/ |
| 5 | Child rearing/ |
| 7 | child parent relation/ |
| 8 | father child relation/ |
| 9 | mother child relation/ |
| A: 1 OR 2 OR 3 OR 4 OR 5 OR 7 OR 8 OR 9 | |
| Intervention | |
| 11 | eHealth.ti,ab |
| 12 | e-Health.ti,ab |
| 13 | e-therapy.ti,ab |
| 14 | e-intervention.ti,ab |
| 19 | ((online or digital or web* or internet* or electronic* or self-administered) adj3 (health or therap* or intervention* or program)).ti,ab |
| 20 | Internet/ |
| 21 | web-based intervention/ |
| 23 | Digital Technology/ |
| B: 11 OR 12 OR 13 OR 14 OR 19 OR 20 OR 21 OR 23 | |
| Outcome | |
| 25 | experienc*.ti,ab |
| 26 | accept*.ti,ab |
| 27 | satisfaction.ti,ab |
| 28 | perspective*.ti,ab |
| 29 | usability.ti,ab |
| 30 | perception*.ti,ab |
| 31 | attitude*.ti,ab |
| 33 | experience/ |
| 34 | patient satisfaction/ |
| C: 25 OR 26 OR 27 OR 28 OR 29 OR 30 OR 31 OR 33 OR 34 | |
| Study | |
| 37 | interview*.ti,ab |
| 38 | Qualitative*.ti,ab |
| 39 | focus group*.ti,ab |
| 40 | mixed method*.ti,ab |
| 41 | feasibility.ti,ab |
| 42 | interview/ |
| 43 | exp qualitative research/ |
| 45 | feasibility study/ |
| D: 36 OR 37 OR 38 OR 39 OR 40 OR 41 OR 42 OR 43 OR 45 | |
| E: A AND B AND C AND D | |
| English language and year (2012) limits applied to E | |

CINAHL

| # | Key words or Subject Heading |
| --- | --- |
| Population | |
| 1 | TI famil* OR AB famil* |
| 2 | TI child* OR AB child* |
| 3 | TI parent* OR AB parent* |
| 4 | (MH "Family") |
| 5 | (MH "child rearing") |
| 6 | (MH "Parent-Child Relations+") |
| A: 1 OR 2 OR 3 OR 4 OR 5 OR 6 | |
| Intervention | |
| 11 | TI eHealth OR AB eHealth |
| 12 | TI e-Health OR AB e-Health |
| 13 | TI e-therapy OR AB e-therapy |
| 14 | TI e-intervention OR AB e-intervention |
| 19 | TI ((online or digital or web* or internet* or electronic* or self-administered) N3 (health or therap* or intervention* or program)) OR AB TI ((online or digital or web* or internet* or electronic* or self-administered) N3 (health or therap* or intervention* or program)) |
| 20 | (MH "Internet") |
| 21 | (MH "Internet-Based Intervention") |
| 23 | (MH "Digital Technology") |
| B: 11 OR 12 OR 13 OR 14 OR 19 OR 20 OR 21 OR 23 | |
| Outcome | |
| 25 | TI experienc* OR AB experienc* |
| 26 | TI accept* OR AB accept* |
| 27 | TI satisfaction OR AB satisfaction |
| 28 | TI perspective* OR AB perspective* |
| 29 | TI usability OR AB usability |
| 30 | TI perception* OR AB perception* |
| 31 | TI attitude* OR AB attitude* |
| 33 | (MH "Patient Satisfaction+") |
| 34 | (MH "Consumer Satisfaction") |
| C: 25 OR 26 OR 27 OR 28 OR 29 OR 30 OR 31 OR 33 OR 34 | |
| Study | |
| 36 | TI interview* OR AB interview* |
| 37 | TI Qualitative* OR AB Qualitative* |
| 38 | TI focus group* OR AB focus group* |
| 39 | TI mixed method* OR AB mixed method* |
| 40 | TI feasibility OR AB feasibility |
| 41 | (MH "Interviews+") |
| 42 | (MH "Qualitative Studies") |
| 43 | (MH "Focus Groups") |
| D: 36 OR 37 OR 38 OR 39 OR 40 OR 41 OR 42 OR 43 | |
| E: A AND B AND C AND D | |
| English language and year (2012) limits applied to E | |

Scopus

| # | Key words or Subject Heading |
| --- | --- |
| Population | |
| 1 | TITLE-ABS (famil*) |
| 2 | TITLE-ABS (child*) |
| 3 | TITLE-ABS (parent*) |
| A: 1 OR 2 OR 3 | |
| Intervention | |
| 11 | TITLE-ABS (eHealth) |
| 16 | TITLE-ABS ((online or digital or web* or internet* or electronic* or self-administered) W/3 (health or therap* or intervention* or program)) |
| 17 | TITLE-ABS (e-Health) |
| 18 | TITLE-ABS (e-therapy) |
| 19 | TITLE-ABS (e-intervention) |
| B: 11 OR 16 OR 17 OR 18 OR 19 | |
| Outcome | |
| 25 | TITLE-ABS (experienc*) |
| 26 | TITLE-ABS (accept*) |
| 27 | TITLE-ABS (satisfaction) |
| 28 | TITLE-ABS (perspective*) |
| 29 | TITLE-ABS (usability) |
| 30 | TITLE-ABS (perception*) |
| 31 | TITLE-ABS (attitude*) |
| C: 25 OR 26 OR 27 OR 28 OR 29 OR 30 OR 31 | |
| Study | |
| 36 | TITLE-ABS (interview*) |
| 37 | TITLE-ABS (qualitative*) |
| 38 | TITLE-ABS (focus AND group*) |
| 39 | TITLE-ABS (mixed AND method*) |
| 40 | TITLE-ABS (feasibility) |
| D: 36 OR 37 OR 38 OR 39 OR 40 | |
| E: A AND B AND C AND D | |
| Year (2012) and English language limit applied to E | |
