# Supplementary material for: Families’ Experiences With Family-Focused Web-Based Interventions for Improving Health: Qualitative Systematic Literature Review
Source: J Med Internet Res. 2025 Jan 30;27:e58774. doi: 10.2196/58774 (PMC11826954; doi:10.2196/58774)
Supplement: Multimedia Appendix 3 [file jmir_v27i1e58774_app3.docx]

## Multimedia Appendix 3 – Themes or subthemes, categories, or codes reported in the results section of included studies

| Study | Themes |
| --- | --- |
| Guagliano et al [1], 2019 | - Findings related to recruitment and retention:   - Children trying to convey what FRESH was to parents   - Participation would be time consuming   - Lack of confidence for physical activity   - Reluctance to being measured - Findings related to intervention feasibility, acceptability, fidelity, and optimisation:   - Feasibility and acceptability of FRESH   - Family time   - FRESH website   - Rewards |
| Hatfield et al [2], 2018 | - Taking action to overcome inertia - New insights that led to clear plans for the future - Adolescent empowerment through strengths focus - Having a champion to guide the way |
| Jogova et al [3], 2013 | - Strengths   - Program - Weaknesses   - Privacy and confidentiality   - Sensitivity   - BMI-discussion   - Age-appropriate   - Target audience   - Appeal   - Psychology   - Justification |
| Bevan Jones et al [4], 2020 | - Design features   - Overall design, navigation, and ease of use   - Interactive elements and personalization   - Illustrations and animations - Sections and content   - Language, tone, and amount of information   - Personal stories   - Sections - Integration and context   - Targeted versus universal   - Use with families, carers, and friends   - Integration with services |
| Khan et al [5], 2021 | - Clinician perceptions of and contribution to recruitment - Perceptions of the ORBIT organisation - Expectations of role of the therapist - Parental persuasiveness - Busy lives - High motivation levels |
| Lalloo et al [6], 2022 | - Motivation for program use among youth - Motivating factors for continued program use   - Tailoring for age group   - Resource in moments of pain   - Caregiver encouragement   - Appealing design   - Ease of finding information   - Ability to tract symptoms over time - Rationale for program recommendation among caregivers - Exploring reasons for low engagement - Differential engagement with program components - Promotion of disease self-management behaviors - Facilitation of SCD self-management   - Availability of peer support   - Practical goal setting support   - Library education feature   - Sharing within family unit   - Symptom monitoring and feedback - Influence of the pandemic on self-management |
| Lenhard et al [7], 2016 | - Autonomy   - Self-efficacy   - Flexibility   - Secure self-disclosure - Support   - Clinician support   - Parental support   - Identification & normalization |
| Muller et al [8], 2024 | - Social support factors   - Professional support   - Community support - Family characteristics and lifestyle   - Competing priorities   - Condition severity   - Prior experience - Program design factors   - Perceived usefulness   - Ease-of-use |
| Murray et al [9], 2022 | Intervention components (skills or lessons)   - Cognitive and relaxation strategies helped adolescents and parents cope with stress - Cognitive and relaxation strategies helped adolescents cope with pain during recovery - Strategies for improving sleep were beneficial before surgery and during recovery - Activity pacing and goal setting used to gradually return to regular activities - Parents valued strategies to encourage self-care   Program structure   - Families found narratives relatable and validating - Families appreciated flexibility of online program   Suggestions/improvements   - Rethink timing and reduce length of first postsurgical lesson - Reduce repetitive lesson content - Enhance program accessibility and interactivity |
| Nieto et al [10], 2019 | Positive aspects   - Relaxation technique code - Giving less importance to pain code - Coping strategies code - Distraction techniques code - Pain reduction code - Communication techniques code   Negative aspects   - No pain reduction code |
| Nieto et al [11], 2019 | - Satisfaction with DARWeb - Burden - Pain perception and skills - Satisfaction with online interventions |
| Nieto et al [12], 2015 | - Satisfaction with DARWeb - Ideas for improving DARWeb - Burden - Pain perception and skills - General perceptions about online interventions |
| O’Sullivan et al [13], 2018 | - Independent self-management - Acquiring JIA knowledge and skills - Unique challenges of JIA in Ireland - Views on web-based approach to self-management - Gaining understanding through social support |
| Palermo et al [14], 2018 | N/A |
| Sonney et al [15], 2020 | - User engagement - Acceptability and usability - Balancing routines and burden |
| Stasiak et al [16], 2018 | N/A |
| Stinson et al [17], 2015 | - Design aesthetics   - Layout   - Navigation   - Visual assets   - Visual appeal - Content   - Completeness   - Understandability   - Quality and credibility   - Relevance - Functionality and features - Sociability - Desire to use program in the future |
| Thompson et al [18], 2019 | N/A |
| Thorén et al [19], 2021 | - A transformative journey of lifestyle changes for the whole family   - Parental awareness of needs for change   - Introducing new routines   - Family mission with some battles   - Feelings of more or less support |
| Wade et al [20], 2021 | N/A |
| Wade et al [21], 2017 | N/A |
| Yuen et al [22], 2016 | - Attractiveness - Controllability - Efficiency - Helpfulness - Learnability |
| Simonsson et al [23], 2021 | - Support can come in different shapes   - Support from the therapist despite distance   - Finding support within the family   - Finding support in the format - Self-responsibility can be empowering as well as distressing   - Flexibility and empowerment   - Distress as a consequence of treatment - Acquiring new skills and treatment effects   - Learning and using new skills   - Benefits in everyday life |
| Lee et al [24], 2023 | N/A |
| Andersson et al [25], 2024 | - Opportunities or barriers to engaging in treatment   - Accepting the BA model is important to engaging in treatment   - The therapist is important in many ways   - Self-guided is not for everyone - Parental involvement is valued and welcomed   - Parents acquire new skills   - The treatment helps find common ground   - Parents’ need for support |
| Connan et al [26], 2019 | - Attributes of learner-centered design - Module content - Design aesthetic - Learner experience |
| Khan et al [27], 2022 | - Mechanisms of impact - Intervention outcomes - Online treatment content |
| Hatfield et al [28], 2017 | N/A |

N/A: not applicable

### References

1. Guagliano JM, Brown HE, Coombes E, Hughes C, Jones AP, Morton KL, Wilson EC, van Sluijs EM. The development and feasibility of a randomised family-based physical activity promotion intervention: the Families Reporting Every Step to Health (FRESH) study. Pilot Feasibility Stud 2019 Feb 9; 5(1):21

2. Hatfield M, Falkmer M, Falkmer T, Ciccarelli M. Process evaluation of the BOOST-A™ transition planning program for adolescents on the autism spectrum: a strengths-based approach. J Autism Dev Disord 2018 Feb 10; 48(2):377-88

3. Jogova M, Song JE, Campbell AC, Warbuton D, Warshawski T, Chanoine JP. Process evaluation of the Living Green, Healthy and Thrifty (LiGHT) web-based child obesity management program: combining health promotion with ecology and economy. Can J Diabetes 2013 Apr; 37(2):72-81

4. Bevan Jones R, Thapar A, Rice F, Mars B, Agha SS, Smith D, Merry S, Stallard P, Thapar AK, Jones I, Simpson SA. A digital intervention for adolescent depression (MoodHwb): mixed methods feasibility evaluation. JMIR Ment Health 2020 Jul 17; 7(7):e14536

5. Khan K, Hollis C, Hall CL, Murray E, Davies EB, Andrén P, Mataix-Cols D, Murphy T, Glazebrook C. Fidelity of delivery and contextual factors influencing children's level of engagement: process evaluation of the online remote behavioral intervention for tics trial. J Med Internet Res 2021 Jun 21; 23(6):e25470

6. Lalloo C, Nishat F, Zempsky W, Bakshi N, Badawy S, Ko YJ, Dampier C, Stinson J, Palermo TM. Characterizing user engagement with a digital intervention for pain self-management among youth with sickle cell disease and their caregivers: subanalysis of a randomized controlled trial. J Med Internet Res 2022 Aug 30; 24(8):e40096

7. Lenhard F, Vigerland S, Engberg H, Hallberg A, Thermaenius H, Serlachius E. "On my own, but not alone" - adolescents' experiences of internet-delivered cognitive behavior therapy for obsessive-compulsive disorder. PLoS One 2016 Oct 6; 11(10):e0164311

8. Muller JL, Tomlin L, March S, Jackson B, Budden T, Law KH, Dimmock JA. Understanding parent perspectives on engagement with online youth-focused mental health programs. Psychol Health 2024 May 27; 39(5):613-30

9. Murray CB, Bartlett A, Meyyappan A, Palermo TM, Aaron R, Rabbitts J. A pilot feasibility and acceptability study of an internet-delivered psychosocial intervention to reduce postoperative pain in adolescents undergoing spinal fusion. Can J Pain 2022 Apr 13; 6(1):12-23

10. Nieto R, Boixadós M, Hernández E, Beneitez I, Huguet A, McGrath P. Quantitative and qualitative testing of DARWeb: an online self-guided intervention for children with functional abdominal pain and their parents. Health Informatics J 2019 Dec 04; 25(4):1511-27

11. Nieto R, Boixadós M, Ruiz G, Hernández E, Huguet A. Effects and experiences of families following a web-based psychosocial intervention for children with functional abdominal pain and their parents: a mixed-methods pilot randomized controlled trial. J Pain Res 2019; 12:3395-412

12. Nieto R, Hernández E, Boixadós M, Huguet A, Beneitez I, McGrath P. Testing the feasibility of DARWeb: an online intervention for children with functional abdominal pain and their parents. Clin J Pain 2015 Jun; 31(6):493-503

13. O'Sullivan G, O'Higgins S, Caes L, Saetes S, McGuire BE, Stinson J. Self-management needs of Irish adolescents with Juvenile Idiopathic Arthritis (JIA): how can a Canadian web-based programme meet these needs?. Pediatr Rheumatol Online J 2018 Nov 08; 16(1):68

14. Palermo TM, Dudeney J, Santanelli JP, Carletti A, Zempsky WT. Feasibility and acceptability of internet-delivered cognitive behavioral therapy for chronic pain in adolescents with sickle cell disease and their parents. J Pediatr Hematol Oncol 2018 Mar; 40(2):122-7

15. Sonney JT, Thompson HJ, Landis CA, Pike KC, Chen ML, Garrison MM, Ward TM. Sleep intervention for children with asthma and their parents (SKIP Study): a novel web-based shared management pilot study. J Clin Sleep Med 2020 Jun 15; 16(6):925-36

16. Stasiak K, Merry SN, Frampton C, Moor S. Delivering solid treatments on shaky ground: feasibility study of an online therapy for child anxiety in the aftermath of a natural disaster. Psychother Res 2018 Jul; 28(4):643-53

17. Stinson J, Gupta A, Dupuis F, Dick B, Laverdière C, LeMay S, Sung L, Dettmer E, Gomer S, Lober J, Chan CY. Usability testing of an online self-management program for adolescents with cancer. J Pediatr Oncol Nurs 2015; 32(2):70-82

18. Thompson D, Callender C, Gonynor C, Cullen KW, Redondo MJ, Butler A, Anderson BJ. Using relational agents to promote family communication around type 1 diabetes self-management in the diabetes family teamwork online intervention: longitudinal pilot study. J Med Internet Res 2019 Sept 13; 21(9):e15318

19. Thorén A, Janson A, Persson M. 'Now she prefers jeans, like everyone else…' - parents' experiences of group- and web-based treatment of children's obesity. Acta Paediatr 2021 Jun 15; 110(6):1869-79

20. Wade SL, Jones KM, Corti C, Adlam AR, Limond J, Bardoni A, Gies LM. Adapting intervention approaches to new contexts: three case studies of international adaptation of the Teen Online Problem Solving (TOPS) program. Rehabilitation Psychology 2021 Nov; 66(4):356-65

21. Wade SL, Narad ME, Kingery KM, Taylor HG, Stancin T, Kirkwood MW, Yeates KO. Teen online problem solving for teens with traumatic brain injury: rationale, methods, and preliminary feasibility of a teen only intervention. Rehabil Psychol 2017 Aug; 62(3):290-9

22. Yuen EK, Gros K, Welsh KE, McCauley J, Resnick HS, Danielson CK, Price M, Ruggiero KJ. Development and preliminary testing of a web-based, self-help application for disaster-affected families. Health Informatics J 2016 Sept 26; 22(3):659-75

23. Simonsson O, Engberg H, Bjureberg J, Ljótsson B, Stensils J, Sahlin H, Hellner C. Experiences of an online treatment for adolescents with nonsuicidal self-injury and their caregivers: qualitative study. JMIR Form Res 2021 Jul 23; 5(7):e17910

24. Lee HY, Xiong S, Sur A, Khang T, Vue B, Culhane-Pera KA, Pergament S, Torres MB, Koopmeiners JS, Desai J. Evaluating human papillomavirus eHealth in Hmong adolescents to promote vaccinations: pilot feasibility study. JMIR Form Res 2023 Jun 20; 7:e38388

25. Andersson R, Vigerland S, Ahlen J, Widström H, Unger I, Serlachius E, Engberg H. "Therapy without a therapist?" the experiences of adolescents and their parents of online behavioural activation for depression with and without therapist support. Eur Child Adolesc Psychiatry 2024 Jan 17; 33(1):105-14

26. Connan V, Marcon MA, Mahmud FH, Assor E, Martincevic I, Bandsma RH, Vresk L, Walsh CM. Online education for gluten-free diet teaching: development and usability testing of an e-learning module for children with concurrent celiac disease and type 1 diabetes. Pediatr Diabetes 2019 May 10; 20(3):293-303

27. Khan K, Hollis C, Hall CL, Davies EB, Murray E, Andrén P, Mataix-Cols D, Murphy T, Glazebrook C. Factors influencing the efficacy of an online behavioural intervention for children and young people with tics: process evaluation of a randomised controlled trial. J Behav Cogn Ther 2022 Sept; 32(3):197-206

28. Hatfield M, Murray N, Ciccarelli M, Falkmer T, Falkmer M. Pilot of the BOOST-A™: an online transition planning program for adolescents with autism. Aust Occup Ther J 2017 Dec 19; 64(6):448-56
